# Post-Antibiotic Effect of Ampicillin and Levofloxacin to *Escherichia coli* and *Staphylococcus aureus* Based on Microscopic Imaging Analysis

**DOI:** 10.3390/antibiotics9080458

**Published:** 2020-07-29

**Authors:** Farjana Hanif Proma, Mohiuddin Khan Shourav, Jungil Choi

**Affiliations:** 1 Department of Integrative Biomedical Science and Engineering, Graduate School, Kookmin University, 77 Jeongneung-ro, Seongbuk-gu, Seoul 02707, Korea; farjana94@kookmin.ac.kr; 2 Department of Mechanical Engineering, Graduate School, Kookmin University, 77 Jeongneung-ro, Seongbuk-gu, Seoul 02707, Korea; khan@kookmin.ac.kr; 3 School of Mechanical Engineering, Kookmin University, 77 Jeongneung-ro, Seongbuk-gu, Seoul 02707, Korea

**Keywords:** post-antibiotic effect, antibiotic, microscopic imaging analysis, *Escherichia coli*, *Staphylococcus aureus*

## Abstract

Post-antibiotic effect (PAE) is the continued suppression of bacterial growth following a limited exposure to an antimicrobial agent. The presence of PAE needs consequential consideration in designing antibiotic dosage regimens. To understand the behavior of bacteria, PAE provides information on how long antibiotics are applied to the bacteria. Conventional methods of measuring PAE depend on population detection and have limitations for understanding the individual behavior of bacteria. To observe the PAE, we utilized an imaging technique with the use of microscopy. Here, we discuss the microscopic image analysis system we used to study the PAE at a single-colony level. The size and number of colonies of bacteria were measured prior to and following antibiotic removal. We could count a single colony, see the development of the settlement prior to and following exposure of antibiotics and track the colony by microscopy according to the incubation time and the image processed by our own image processing program. The PAE of antibiotics was quantified by comparing bacteria size and number based on their exposure time. In our study, we discovered that the longer exposure of antibiotics causes the bacteria to be suppressed—even after washing the antibiotics from the solution. This finding suggests that microscopic imaging detection provides a new method for understanding PAE. In addition, the behavior of the cell in response to drugs and chemicals and their removal can be examined with the use of single colony analysis.

## 1. Introduction

Today, a re-emergence of infectious diseases has been observed that is due to both antibiotic resistance and tolerance. There are two main ways to combat this risk: first, the development of novel antibiotics and, second, the utilization of existing drugs more effectively to lessen the change of resistance emergence [1,2]. The development of a new drug is a time- and resource-intensive process. Moreover, pharmaceutical companies are not financially incentivized to develop these types of drugs. Therefore, it is of increasing paramount to understand the population dynamics underlying different bacterial survival mechanisms and utilize this knowledge to design better antibiotic treatment protocols [3,4,5]. A clear idea on a prevalent phenomenon known as the post-antibiotic effect (PAE) will be provided by this study, which implies the transient suppression of bacterial magnification after antibiotic treatment. Customarily, PAE has been determined by counting a viable cell number and measuring turbidity of culture media. A conventional method that cannot measure single colony bacteria measures the population level because of a detection limit.

PAE is the continued suppression of bacterial growth following a limited exposure to an antimicrobial agent [6]. The effect of the inhibition of the antimicrobial agent may last after drug levels are no longer detectable. Thus, the knowledge of the duration of PAE may be essential in showing the schedules of the dose for the treatment of infections; hence, antibiotics may be ineffective during PAE [7,8]. Therefore, it may be considered prudent to perceive the duration of PAE for different microorganisms and antibiotic combinations. A viable count method is among the most commonly accepted means for measuring PAE, although this is a population measurement procedure, which includes a long incubation step. In a viable count method, it is possible to generate inaccurate findings with some antibiotics, notably β-lactams [9]. There is another method for counting PAE namely, bioluminescence, which is faster than the viable count method. On the contrary, a poor correlation between bioluminescence and the viable count method has been shown in recent study [10]. The biggest disadvantage of this technique is its laborious nature, leading to the development and application of the other techniques to the study of PAE, for example, electrical impedance, bacterial morphology, infrared spectroscopy, radiometry, electrical counting, fluorimeter and spectrophotometer [11,12]. In any PAE measurement method, it is a must to separate antibiotics from the cells’ environment for the recovery of cells. This is primarily achieved through centrifugation, filtration or dilution of both cells and antibiotics, with dilution of the latter to a concentration below an inhibitory level. As an alternative to PAE determination based on single colony count procedures, various authors have investigated the possibility that microscopic imaging methods may be utilized to monitor more quickly the regrowth of organisms after antibiotic exposure [13,14]. Therefore, single colony analysis methods were utilized to determine PAE of ampicillin and levofloxacin to *E. coli* and *S. aureus*.

In our system, we observed PAE in a single colony level by microscopic imaging analysis [15,16,17]. We could recognize what number of states can endure the distinction of settlement simultaneously as well as the size of the colony. On the off chance that we exposed it for a long time, the development rate became less. In other frameworks for estimation, PAE resembled the estimation of the turbidity or count number of cells on the agar plate. In addition, by doing these techniques, we do not have a clear idea what number of bacteria survived and divided again. *Escherichia coli*—a widely studied bacterium—is mostly found in urinary tract infections [18,19]. *Staphylococcus aureus*, considered as one of the major human pathogens, caused a wide range of clinical infections, such as bacteremia, infective endocarditis, and osteoarticular, skin and soft tissue infections [20] We had conducted our technique with ampicillin and levofloxacin against *S. aureus* and *E. coli*. This was the confinement of population analysis.

## 2. Results

### 2.1. PAE of Ampicillin and Levofloxacin to E. coli ATCC 25922

**Figure 1:**
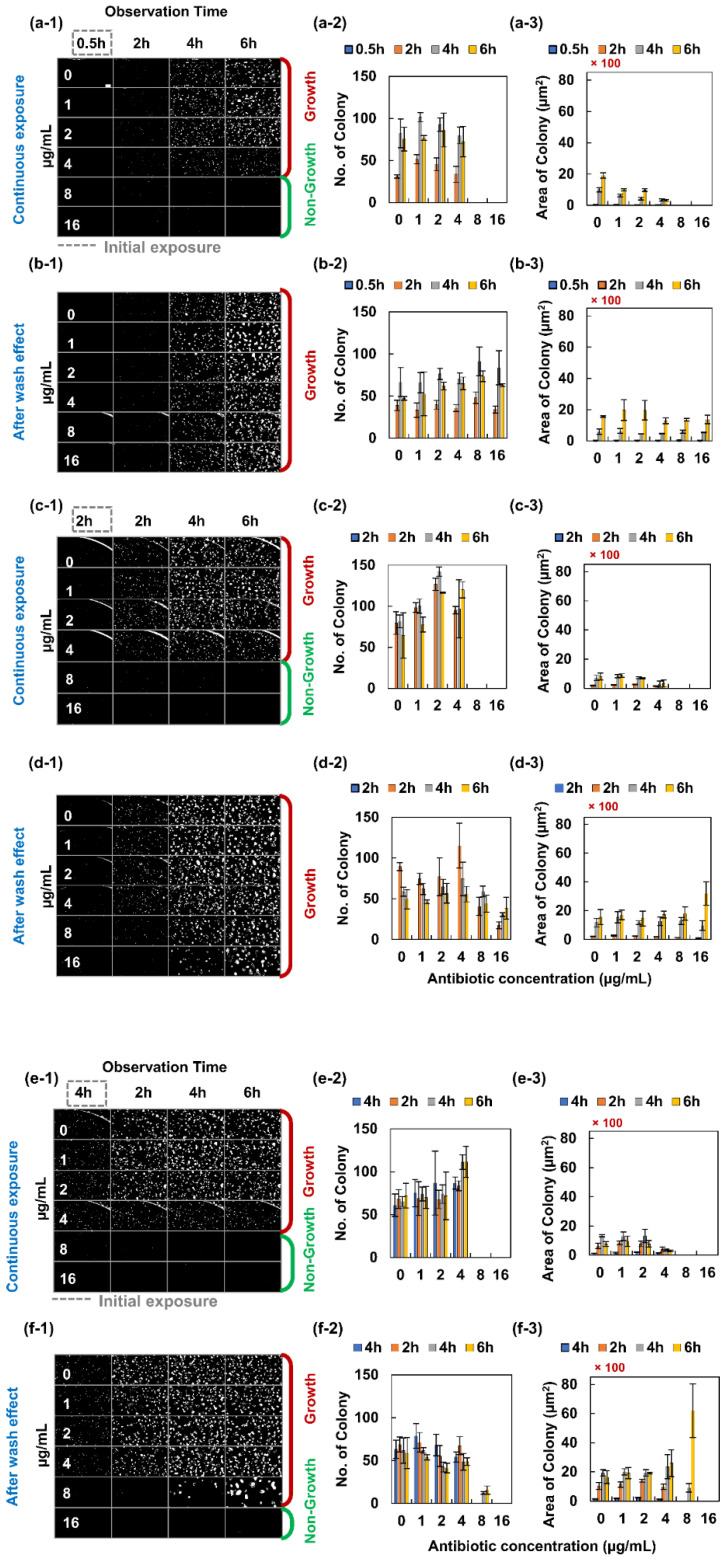
Post-antibiotic effect (PAE) of ampicillin to *E. coli*. *Escherichia coli* was initially exposed to ampicillin for 0.5, 2 and 4 h. (**a**,**b**), (**c**,**d**) and (**e**,**f**) represent the PAE analysis of 0.5, 2 and 4 h, respectively. (**a**,**c**,**e**) shows the image analysis for 0.5-, 2- and 4-h exposure to ampicillin following 3 more observations with a 2-h interval. Nonetheless, (**b**,**d**,**f**) indicates the examination of PAE following initial exposure. The colony of bacteria was measured and then plotted as shown in the second position of all the existing alphabets in figure. In addition, the colony area was calculated and shown in the third position of all the alphabets in figure. The scale bar represents 100 µm.

**Figure 2:**
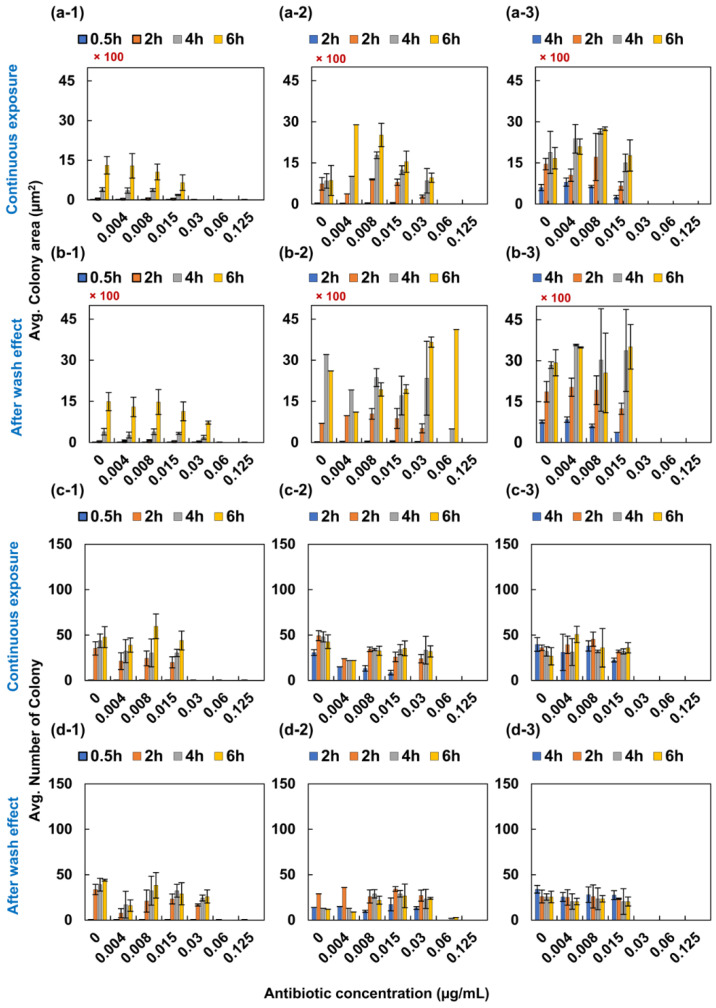
PAE of levofloxacin to *E. coli*. *Escherichia coli* was initially exposed to levofloxacin for 0.5, 2 and 4 h. (**a**-**1**–**a**-**3**) displays the image analysis of colony area and (**c**-**1**–**d**-**3**) displays the image analysis of the number of colonies for 0.5-, 2- and 4-h exposure to levofloxacin following 3 more observations with a 2-h interval. Nonetheless, (**b**-**1**–**b**-**3**) displays the analysis of PAE following initial exposure. The colony of bacteria was calculated and plotted as shown in the c and d positions of figure.

**Figure 3:**
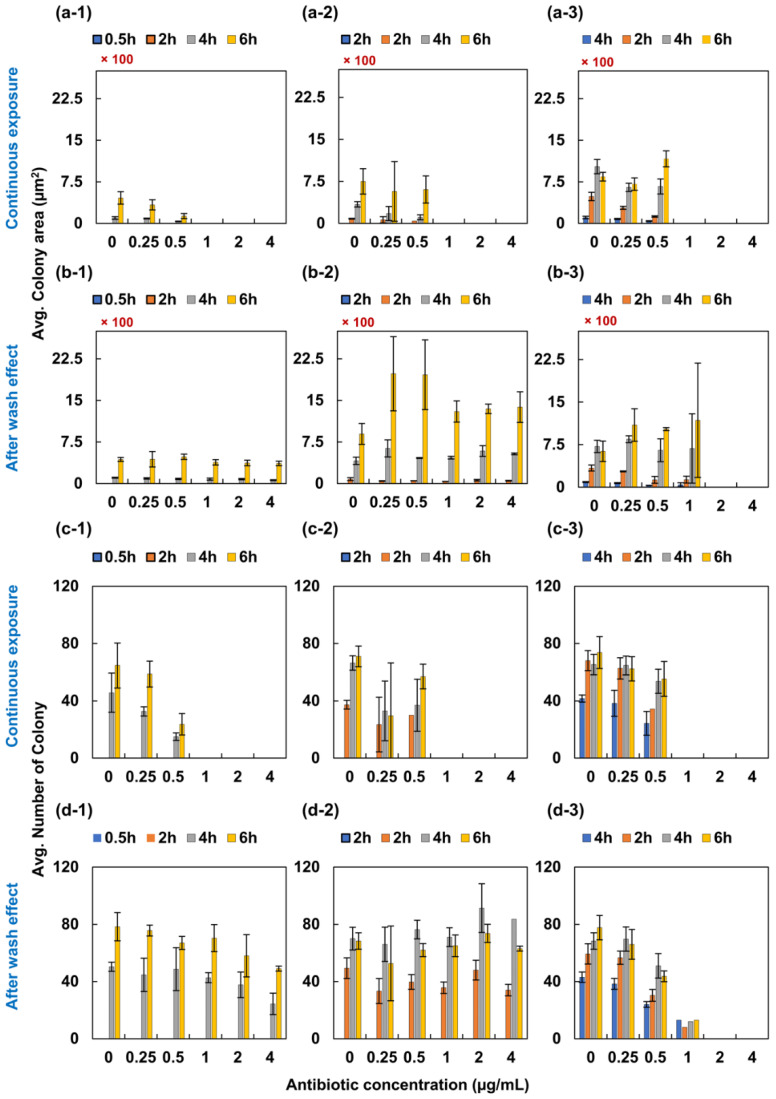
PAE of ampicillin to *S. aureus*. *Staphylococcus aureus* was initially exposed to ampicillin for 0.5, 2 and 4 h. (**a**-**1**–**a**-**3**) displays the image analysis of colony area and (**c**-**1**–**d**-**3**) displays the image analysis of the number of colonies for 0.5-, 2- and 4-h exposure to ampicillin following 3 more observation with a 2-h interval. Nonetheless, (**b**-**1**–**b**-**3**) shows the analysis of PAE following initial exposure. The colony of bacteria was measured and plotted as shown in the c and d positions in figure.

*E. coli* ATCC 25922 was exposed to ampicillin initially in 0.5, 2 and 4 h, respectively and washed to remove the antimicrobial agent. The washed bacterial samples were observed after 2, 4 and 6 h to determine its PAE. In Figure 1a,c,e, *E. coli* was continually exposed to ampicillin and observed in a 0.5-h, 2-h and 4-h and 2-h interval for 6 h. Under this condition, the bacteria’s colony area was observed under a microscope. The number and area of colonies were measured using image analysis and plotted against ampicillin concentration in the solutions (Figure 1(a-2,a-3)). It is noted from the figure that the minimum inhibitory concentration (MIC) value of *E. coli* against ampicillin was 8 µg/mL. The number of colonies were counted and then plotted in Figure 1c. There were no colonies at 8 and 16 µg/mL (Figure 1a,c,e).

Then, the antibiotic was washed after 0.5 h (Figure 1b). There were colonies at all the concentrations of the antibiotics, giving us an insight that following the removal of the antibiotics from the bacteria; the bacteria start to grow again. Here the ampicillin served as a bacteriostatic antibiotic. Hence, we had discovered from this analysis that, following a 0.5-h exposure to antibiotics, PAE of ampicillin to *E. coli* was not significant and bacteria may grow back again. In addition, *E. coli* was exposed to ampicillin for 2 and 4 h as well. The analysis is shown in Figure 1d,f, respectively. We observed similar characteristics for a 2-h exposure to the bacteria. Bacteria grew back again following the removal of antibiotics. At the highest concentration of 16 µg/mL, the number of colonies decreased, but the average size of colony increased. Nonetheless, for a 4-h initial exposure, bacteria did not grow back at 16 µg/mL—although we observed the number of colonies counted at 8 µg/mL was less than other volumes of antibiotics (Figure 1d,f). In this condition the area of the colony was much higher than the other volume of antibiotic. This was due to the quorum sensing effect of the bacteria [21]. When the number of colonies decreased, the size of the bacteria increased.

However, *E. coli* grew bigger in size when incubated for a longer time. We observed ampicillin exposure to *E. coli* for 0.5, 2 and 4 h. The antibiotics were removed by washing all these experiments and its effect under microscopy was observed for 2, 4 and 6 h, respectively (Figure 1b,d,f). We saw that the size of the colony increases with longer incubation times. From our analysis we also observed that there was no colony at the 0.5- and 2-h initial exposure. In the 4-h initial exposure, PAE was reported by our analysis. The colony area did not grow much at 4-h initial exposure—even after the removal of the ampicillin.

In case of levofloxacin to *E. coli*, the MIC value of continuous exposure to *E. coli* was in the range of 0.03 to 0.06 µg/mL (Figure 2a,c). It was in the quality control range from the Clinical and Laboratory Standards Institute (CLSI) [22]. When we removed antibiotics from the testing well, the MIC value increased to 0.06 and 0.125 µg/mL in 0.5- and 2-h exposure cases, respectively (Figure 2b,d). However, in case of 4-h exposure, the MIC value remained as 0.03 µg/mL.

### 2.2. PAE of Ampicillin and Levofloxacin to S. aureus ATCC 29213

*S. aureus* ATCC 29213 was initially exposed to ampicillin and levofloxacin for 0.5, 2 and 4 h and observed for 6 more h with a 2-h interval. In our study, we split the observation into two categories: one category allowed the bacteria to remain exposed with the antibiotic, and in another category, the sample was washed to observe the effect caused by the initial exposure. The MIC of ampicillin to *S. aureus* was 1 µg/mL during the continuous exposure for 6 h following the initial exposure (Figure 3a,c). Nonetheless, bacteria grew back in all volumes of antibiotics following washing in 0.5- and 2-h initial exposure (Figure 3b,d). It was interesting to note that *S. aureus* did not grow again in 2 and 4 µg/mL after washing at 4-h initial exposure (Figure 3(b-3)). The MIC was found—following the washing—to be 0.5, 0.5 and 0.12 µg/mL for 0.5, 2 and 4 h, respectively.

**Figure 4:**
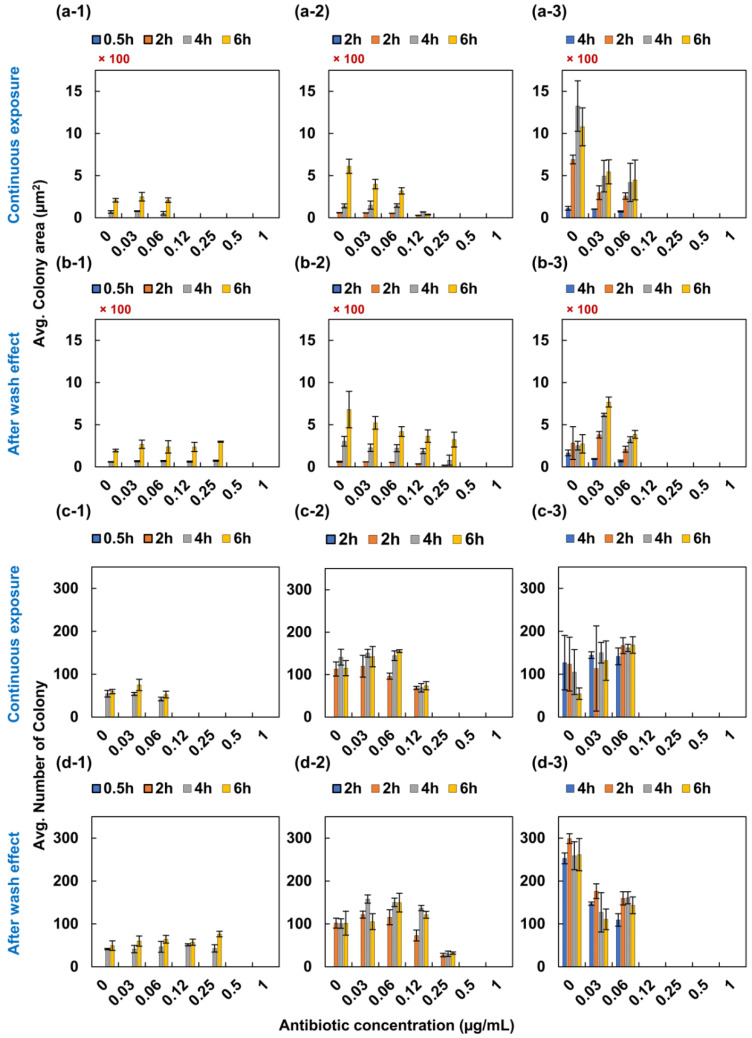
PAE of levofloxacin to *S. aureus*. *Staphylococcus aureus* was initially exposed to levofloxacin for 0.5, 2 and 4 h. (**a**-**1**–**a**-**3**) displays the image analysis of the colony area and (**c**-**1**–**d**-**3**) displays the image analysis of the number of colonies for 0.5-, 2- and 4-h exposure to levofloxacin following 3 more observations with a 2-h interval. However, (**b**-**1**–**b**-**3**) displays the examination of PAE following initial exposure. The colony of bacteria was measured and plotted as shown in the c and d positions in figure.

In case of levofloxacin to *S. aureus*, the MIC value of continuous exposure to *S. aureus* was in the range of 0.12 to 0.25 µg/mL (Figure 4a,c). It was in the quality control range from the CLSI. When we removed antibiotics from the testing well, the MIC value increased to 0.5 µg/mL in 0.5- and 2-h exposure cased, respectively (Figure 4b,d). However, in case of 4-h exposure, the MIC value remained 0.12 µg/mL.

## 3. Discussion

The correct utilization of antimicrobial operators for the treatment of bacterial disease needs information on the weakness of the particular strain to antimicrobial agents. When selecting the right choice, we should estimate PAE, which gives data regarding the activity of antibiotics. PAE is significant for deciding longer dosing interims, reducing the antagonistic effect on life forms, reducing the spread of antimicrobial obstruction and reducing the cost of treatment. PAE determination has been investigated for a long time now. Various techniques have been suggested by several researchers. Among them viable count is the standard technique, which is much laborious to conduct. Likewise, this method needs population growth estimation. There are some other techniques that have been suggested as an alternative to the viable count method. William (2004) et al. proposed a method using a microplate [23], which is close to the viable counting methods as shown in their findings. However, in our study we have conducted a single colony analysis, providing the individual colony’s information by calculating its area and number, which may help us understand the bacteria’s characteristics more intuitively. We designed a microscopic single colony analyzing system for PAE analysis. The number of single colonies can be counted, the development of the settlement can be seen prior to and following expulsion of antibiotics, and the colony is tracked by image processing program according to the incubation time. For most enterococci, penicillin, ampicillin, vancomycin and teicoplanin usually display bacteriostatic activity at clinically achievable concentrations where *E. coli* can become resistant to the antibiotic ampicillin [24].

From the analysis, the bacteria can grow again even following the exposure to antibiotics after relatively short time of exposure from 0.5- to 2-h. However, after 4-h of exposure, the MIC value is same as continuous exposure case. This study may help improvise the antibacterial dose and duration of taking antibiotic. This study could provide us an insight for PAE determination with the use of a single cell analysis method. This technique could be utilized in the facility to enhance PAE study and create a quick decision.

**Figure 5:**
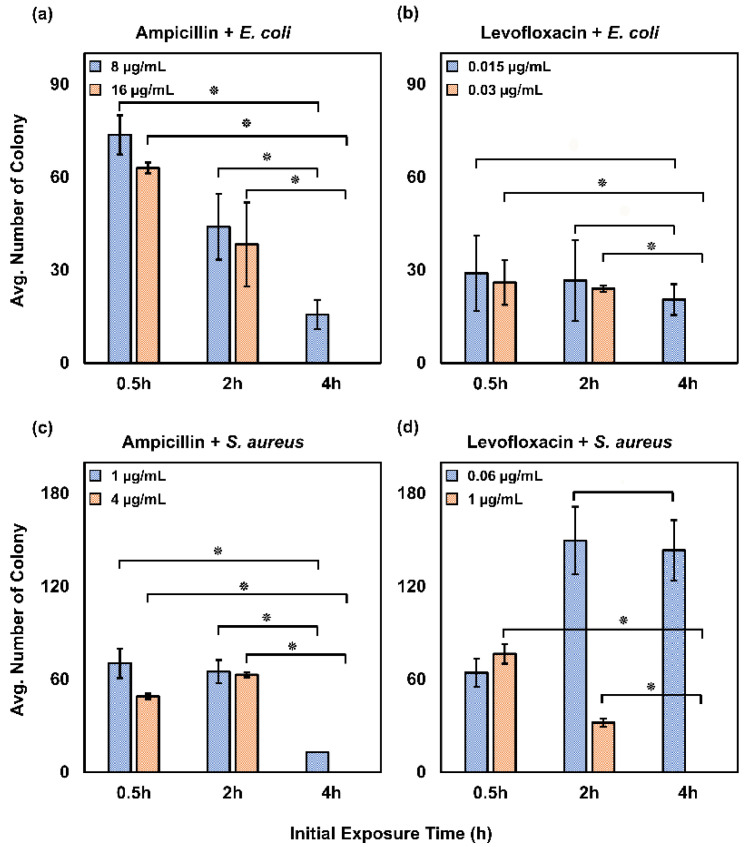
Exposure time depended PAE effect observation. *E. coli* in ampicillin (**a**) and levofloxacin (**b**) respectively. *S. aureus* exposed to ampicillin (**c**) and levofloxacin (**d**) This repeating trend was further justified by student T-test (*p* < 0.05). * indicates the significant difference (*p* ≤ 0.05) of average colony count between the short and long exposure time.

The average of the colony number was plotted against the initial exposure time. We averaged the colony number found in two different concentrations of antibiotics after washing. One of them is the highest level of antibiotic concentration and another one is the colony found at any concentration before the highest concentration of antibiotics. For example, *E. coli* was exposed to ampicillin from 1 to 16 µg/mL concentration; we have observed that the colony grows until 8 µg/mL at 4 h exposure as, in Figure 5, these two concentrations are plotted at different exposure times. Student’s T-test (*p* < 0.05) was performed to demonstrate the significant difference between the exposure time for PAE. * indicates the significant difference (*p* ≤ 0.05) in the average colony count between the short and long exposure time. The highest exposure time in our experiment shows no growth of bacteria after the washing of antibiotics, whereas short exposure times cannot completely eliminate the bacterial growth after removing the antibiotics, as found in our experiments. This result is known as the heterogenetic effect. Heterogenicity is observed during antibiotic exposure to the bacteria [25]. After changing the environment of the bacteria, all the colonies may not survive, even if they are introduced with nutrition. However, it is interesting to note that the surviving cell area starts to increase more than the cell that was exposed to the antibiotics.

There is no direct comparison reported with any standard methods in our study, although this report may open an alternative opportunity for PAE measurement with the use of a single colony imaging technique. Our study was limited to the use of single colony imaging for the observation of the PAE. This study may further be validated by comparing with other standard techniques.

## 4. Materials and Methods

### 4.1. PAE Determination by Single Colony Analysis

We used the microfabricated culture chip system, which was utilized to immobilize bacteria for single colony analysis. Fluid agarose with microbes was conveyed into the channel. The agarose was then cemented to immobilize the bacteria. At that point, the way of life media and antibiotics diffused to where the bacteria were in the agarose matrix, which had a permeable structure sufficient for dispersion to occur. Using a multipipette, the antibiotic and bacteria were administered into wells of a microliter plate as indicated by the grouping of antibiotics. The well remained drug free for the control. Single bacterial cells in the transparent agarose matrix were monitored with the use of a microscope.

**Figure 6:**
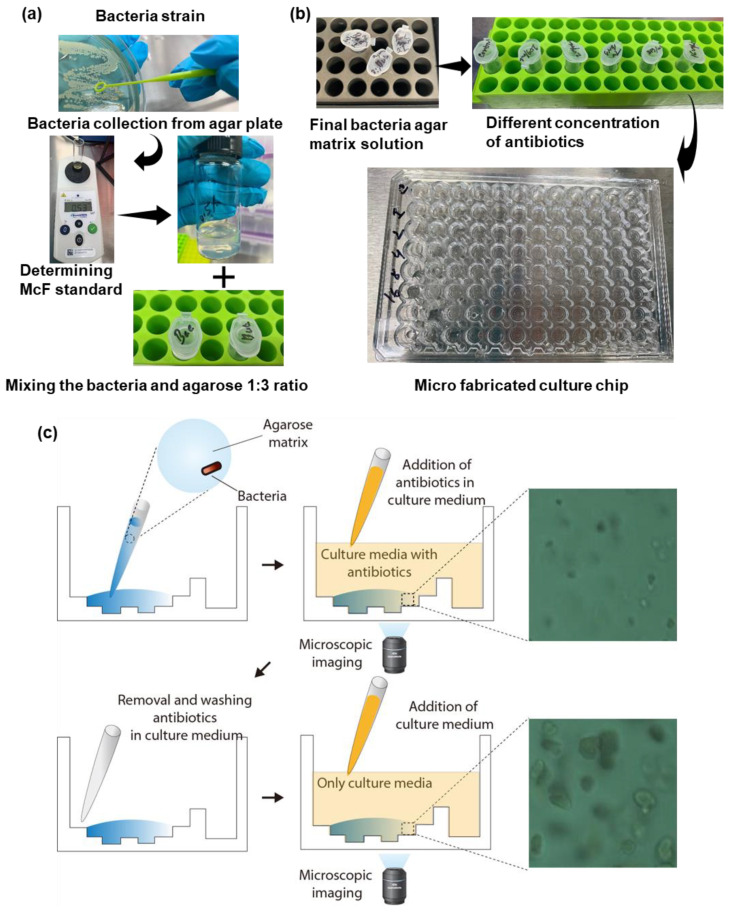
Experimental sample preparation. (**a**) Sample preparation using bacteria and agarose; (**b**) bacteria with antibiotics for PAE observation in the microfabricated culture chip and (**c**) protocol of addition, removal and washing of antibiotics.

Following the experimental plan, the antibiotic was removed, the bacteria with culture media washed five times and bacteria exposed with culture media and incubated again in different hours. Two standard bacteria of the Clinical and Laboratory Standard Institute strains, *E. coli* ATCC 25922 and *S. aureus* ATCC 29213, were tested with ampicillin and levofloxacin to determine their PAE values. The well-organization of microfabricated chip was coordinated with a well stage for high-throughput examination [17]. The microchip was made from microfluidics containing bacteria in agarose, which was shown in Figure 6b, and a well for flexible antibiotic agents and nutrients as shown in Figure 6a. The imaging area was the interface between the fluid medium and the microfluidic channel. The immobilized bacterial cells on bottoms of channels were checked for PAE with the used of time-lapse bright field microscopy provided by QuantaMatrix Inc. For initial exposure, antibiotics in culture media were added to the culture well. Following initial exposure, the antibiotic solution was removed via pipetting and washed by 100 µL of culture media 5 times. After washing, 100 µL of culture media was added for culture of the bacteria (Figure 6c).

### 4.2. Single Colony Analysis by Image Processing

**Figure 7:**
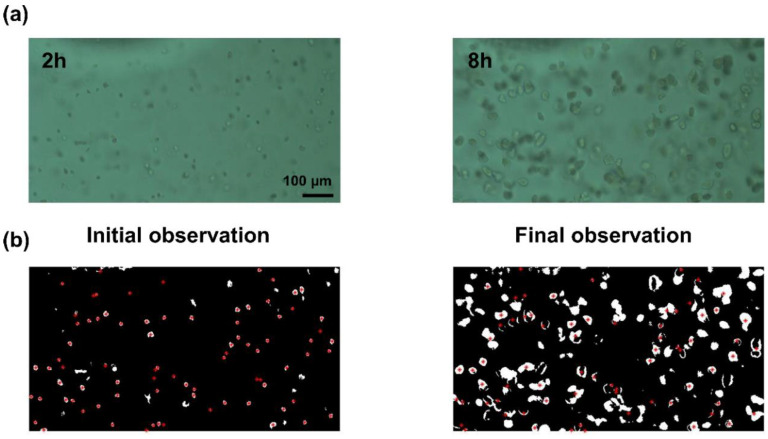
Image processing for observation of the PAE. (**a**) The raw image represents the bacteria solution at various timepoints; (**b**) Tracing and measuring of cell area from initial observation to final observation. The red dot represents the same colony of the observation.

Bacterial cells were imaged under a 20 × microscopic objective lens in a dedicated bacterial cell imaging device. A microfabricated chamber was utilized to contain the bacterial cells. The images were taken using a CMOS camera; the raw images were then processed to determine the minimum inhibitory concentration values of the antibiotics and afterward washed as a PAE. An image processing program was constructed and coded in MATLAB (version 2019, MA, USA), to transform the image data into digital data as shown in Figure 7. For the calculations, RGB images were transformed into gray formatted images. After this, the processed images were modified into binary format images (Figure 7b). The background of each image was distinguished from the bacteria and then eliminated. Then, the background eliminated images were filtered by elimination of the pixels less than 25.

The processing program measured the areas in pixel unit that were occupied by each bacterium cell. A longer incubation time permitted each bacterium cell to grow and occupy more area. Here we have tracked the same colony from its initial observation (red dot in Figure 7b) to the final image so that we can find the development or effect of the cells as shown in Figure 7b. In result, the increasement of the size of colony could be calculated even there are more colony appeared in the final images. The imaging processing program worked well as it measured the sizes of the regions that the bacteria occupied. Following image processing, the area occupied by bacterial growth was measured.

### 4.3. Bacterial Strain and Sample Preparation

We utilized the two CLSI standard strains of *E. coli* (ATCC 25922) and *S. aureus* (ATCC 29213) (MicroBioLogics, Inc. MN, USA). We also utilized ampicillin and levofloxacin antimicrobials in the study (Sigma-Aldrich, MO, USA). We created stock solutions using 25% glycerol (Sigma-Aldrich, MO, United States) and stored them at −70 °C. For the test, we inoculated the stock solution into the Luria–Bertani agar (KisanBio Co., Ltd., Republic of Korea) and incubated it at 37 °C overnight. At that point we chose three to five isolated colonies from the cultured agar plate and transferred them into a tube containing Mueller–Hinton broth (CAMHB, BD Biosciences, CA, USA) medium. We then adjusted the bacterial concentration to 1.5 × 10^8^ CFU/mL and diluted to 5.0 × 10^6^ CFU/mL. Two hundred microliters of bacterial solution was mixed with 0.5% agarose at 37 °C. We pipetted 10 µL of the mixture to the center of the one well of microfabricated culture chip and incubated it at 37 °C. During the imaging time, we utilized motorized microscopic imaging system with 20 X lens from QuantaMatrix, Inc. The imaging analysis program in MATLAB (version 2019, MA, USA) conducted the image processing of the bacterial images.

### 4.4. Fabrication of the Microfabricated Culture Chip

We designed the microfabricated culture chip (96-well format) with the use of the 3D CAD design software (SolidWorks v2014, Dassault Systèmes SolidWorks Corp., Velizy, France) and it was fabricated by an injection molding (NT2–120, Woojin Plaimm, Republic of Korea) of polystyrene (K-RESIN, Chevron Phillips Chemical, TX, USA). Before AST, a 1-min air plasma treatment (CUTE-MP, Femto Science, Hwaseong-si, Korea) was utilized for the hydrophilic treatment of the chip.

### 4.5. Quality Control Test

For the quality control of the overall test, the MIC of ampicillin and levofloxacin were determined with the use of a microdilution technique as shown by records of the CLSI. To identify the lowest concentration needed for a given antibiotic to impede bacterial growth, an indistinguishable number of microorganisms are brought into wells of fluid media containing continually lower groupings of the drug. To decide the MIC for our analysis, we utilize the broth microdilution method.

### 4.6. Antibiotic Removal Validation Test

In our experiment—following the exposure time—we evaluated the antibiotic and washed it five times. By this process no antibiotic stayed with the bacteria. We placed the nutrients of the bacteria in the culture media. To guarantee this experiment’s legitimacy, rhodamine B dye with antibiotic was utilized. Rhodamine B (Sigma-Aldrich, St. Louis, MO, USA) molecular weight (479.02 g/mole) and antibiotic molecular weight (ampicillin 349.4 g/mole and levofloxacin 349.406 g/mole) are the same. We utilize a similar concentration in the microfabricated chip plate. The image is taken with the help of fluorescence microscopy.

## 5. Conclusions

In summary, this paper introduced a microscopic analysis of a single colony for understanding PAE. Immobilizing bacteria through agarose and washing through simple pipetting provided a user-friendly system for studying PAE. Time lapse imaging and image processing provided a new way of analyzing the effect of antibiotics after washing. In the case of *E. coli* and *S. aureus* with ampicillin and levofloxacin, a longer exposure of antibiotics induced a stronger inhibition effect in the bacteria. Microscopic image analysis for PAE could be a new alternative to other established techniques. Direct comparisons with conventional methods could provide new insights into PAE.

## References

[1] Levy S.B., Bonnie M. (2004). Antibacterial resistance worldwide: Causes, challenges and responses. Nat. Med..

[2] Roca I., Akova M., Baquero F., Carlet J., Cavaleri M., Coenen S., Cohen J., Findlay D., Gyssens I., Heure O.E. (2015). The global threat of antimicrobial resistance: Science for intervention. New Microbes New Infect..

[3] Cervera C., van Delden C., Gavaldà J., Welte T., Akova M., Carratalà J. (2014). Multidrug-resistant bacteria in solid organ transplant recipients. Clin. Microbiol. Infect..

[4] Coussement J., Scemla A., Abramowicz D., Nagler E.V., Webster A.C. (2018). Antibiotics for asymptomatic bacteriuria in kidney transplant recipients. Cochrane Database Syst. Rev..

[5] Li B., Webster T.J. (2018). Bacteria antibiotic resistance: New challenges and opportunities for implant-associated orthopedic infections. J. Orthop. Res..

[6] Hanberger H., Nilsson L.E., Kihlström E., Maller R. (1990). Postantibiotic effect of beta-lactam antibiotics on Escherichia coli evaluated by bioluminescence assay of bacterial ATP. Antimicrob. Agents Chemother..

[7] Gudmundsson S., Vogelman B., Craig W.A. (1994). Decreased bactericidal activity during the period of the postantibiotic effect. J. Antimicrob. Chemother..

[8] MacKenzie F.M., Gould I.M. (1993). The post-antibiotic effect. J. Antimicrob. Chemother..

[9] Hanberger H., Nilsson L.E., Maller R., Nilsson M. (1990). Pharmacodynamics of beta-lactam antibiotics on gram-negative bacteria: Initial killing, morphology and postantibiotic effect. Scand. J. Infect. Dis. Suppl..

[10] Hanberger H., Svensson E., Nilsson L.E., Nilsson M. (1995). Control-related effective regrowth time and post-antibiotic effect of meropenem on gram-negative bacteria studied by bioluminescence and viable counts. J. Antimicrob. Chemother..

[11] Fang W. (1996). A novel fluorometric method for evaluation of the postantibiotic effect of antibacterial drugs on mastitis-causing Staphylococcus aureus and Escherichia coli. J. Microbiol. Methods.

[12] Coates A.R.M., Halls G., Hu Y. (2011). Novel classes of antibiotics or more of the same?. Br. J. Pharmacol..

[13] Zahir T., Camacho R., Vitale R., Ruckebusch C., Hofkens J., Fauvart M., Michiels J. (2019). High-throughput time-resolved morphology screening in bacteria reveals phenotypic responses to antibiotics. Commun. Biol..

[14] Libby J.M. (1998). Postantibiotic effect in Escherichia coli determined with real-time metabolic monitoring. Antimicrob. Agents Chemother..

[15] Choi J., Jung Y.G., Kim J., Kim S., Jung Y., Na H., Kwon S. (2013). Rapid antibiotic susceptibility testing by tracking single cell growth in a microfluidic agarose channel system. Lab Chip.

[16] Choi J., Yoo J., Lee M., Kim E.-G., Lee J.S., Lee S., Joo S., Song S.H., Kim E.-C., Lee J.C. (2014). A rapid antimicrobial susceptibility test based on single-cell morphological analysis. Sci. Transl. Med..

[17] Choi J., Jeong H.Y., Lee G.Y., Han S., Han S., Jin B., Lim T., Kim S., Kim D.Y., Kim H.C. (2017). Direct, rapid antimicrobial susceptibility test from positive blood cultures based on microscopic imaging analysis. Sci. Rep..

[18] Kumar Prajapati A. (2019). Urinary Tract Infection in Diabetics. Microbiology of Urinary Tract Infections—Microbial Agents and Predisposing Factors.

[19] Rostkowska O.M., Kuthan R., Burban A., Salińska J., Ciebiera M., Młynarczyk G., Durlik M. (2020). Analysis of Susceptibility to Selected Antibiotics in Klebsiella pneumoniae, Escherichia coli, Enterococcus faecalis and Enterococcus faecium Causing Urinary Tract Infections in Kidney Transplant Recipients over 8 Years: Single-Center Study. Antibiotics.

[20] Tong S.Y.C., Davis J.S., Eichenberger E., Holland T.L., Fowler V.G. (2015). Staphylococcus aureus Infections: Epidemiology, Pathophysiology, Clinical Manifestations, and Management. Clin. Microbiol. Rev..

[21] Chacón J.M., Möbius W., Harcombe W.R. (2018). The spatial and metabolic basis of colony size variation. ISME J..

[22] Limbago B. (2001). M100-S11, Performance standards for antimicrobial susceptibility testing. Clin. Microbiol. Newsl..

[23] Stubbings W.J., Bostock J.M., Ingham E., Chopra I. (2004). Assessment of a microplate method for determining the post-antibiotic effect in Staphylococcus aureus and Escherichia coli. J. Antimicrob. Chemother..

[24] Landman D., Quale J.M. (1997). Management of infections due to resistant enterococci: A review of therapeutic options. J. Antimicrob. Chemother..

[25] Davis K.M., Isberg R.R. (2016). Defining heterogeneity within bacterial populations via single cell approaches. Bioessays.

